# Supplementary material for: The Effect of Various Poly (N-vinylpyrrolidone) (PVP) Polymers on the Crystallization of Flutamide
Source: Pharmaceuticals (Basel). 2022 Aug 6;15(8):971. doi: 10.3390/ph15080971 (PMC9414356; doi:10.3390/ph15080971)
Supplement: Supplementary file 1 [file pharmaceuticals-15-00971-s001.zip › pharmaceuticals-1839043-supplementary.pdf]

The Effect of Various Poly(N-vinylpyrrolidone) (PVP) Polymers on the Crystallization of Flutamide

Dawid Heczko,[1,*] Barbara Hachuła,[2] Paulina Maksym,[3] Kamil Kamiński,[4] Andrzej Zięba,[5] Luiza Orszulak,[2] Marian Paluch,[4] Ewa Kaminska[1,*]

[1] Department of Pharmacognosy and Phytochemistry, Faculty of Pharmaceutical Sciences in Sosnowiec, Medical University of Silesia in Katowice, 41-200 Sosnowiec, Poland
[2] Institute of Chemistry, Faculty of Science and Technology, University of Silesia in Katowice, 40-007 Katowice, Poland
[3] Institute of Material Science, Faculty of Science and Technology, University of Silesia in Katowice, 41-500 Chorzów, Poland
[4] Institute of Physics, Faculty of Science and Technology, University of Silesia in Katowice, 41-500 Chorzów, Poland
[5] Department of Organic Chemistry, Faculty of Pharmaceutical Sciences in Sosnowiec, Medical University of Silesia in Katowice, 41-200 Sosnowiec, Poland

** Corresponding authors: D.H. (dawid.heczko@sum.edu.pl), E.K. (ekaminska@sum.edu.pl)*

Supplementary Materials

Synthesis procedures

PVP has been produced *via* high-pressure (HP) thermally-initiated free-radical polymerization (FRP) according to the procedure previously reported by some of us (0.01-0.15 wt% of AIBN) [1]. In this work, a similar methodology (0.5 wt% of AIBN) was used for PVP-OH except that 2-isopropoxyethanol (100 wt% in respect to monomer) was added to act as a solvent and a chain transfer agent. The polymerization process was conducted at p=250 MPa, T=60 ^{0}C within 3h (see Scheme S1). PVP-OH sample was purified by vacuum evaporation, followed by chloroform dissolution and precipitation in cold diethyl ether. Purification using a chloroform-diethyl ether solvent system was repeated four times. The resulting precipitate was collected by filtration and dried over vacuum to a constant mass.

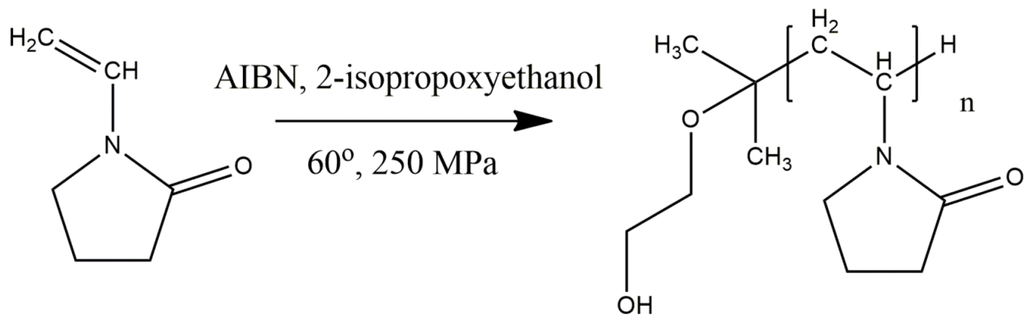

Scheme S1. Synthetic pathway for the production of PVP-OH.

NMR analysis

The PVP-OH structure has been confirmed by ^{1}H and ^{13}C NMR analysis (~ 40 mg in 0.8 mL deuterium oxide). VP conversion was calculated from ^{1}H NMR spectrum ($CDCl_3$) registered for the sample taken from the reaction mixture by comparing the integral areas of the protons of the VP (1H, δ=7.11 ppm) in reference to the methylene protons peaks adjusting to the lactam ring of both VP and PVP (8H, δ=1.85-2.55 ppm). As demonstrated in Figure S1a (^{1}H NMR, D_2O), despite the signals assigned to PVP that is δ=1.42-1.84 (2H, -CH_2-CH-, the main chain); δ=1.80-2.08 (2H, -CH_2-CH_2-CH_2-); 2.10-2.52 (2H, -CH_2-C=O); 3.42-3.90 (1H, -CH_2-CH-, the main chain); 2.90-3.40 (2H, -CH_2-N-), also the hydroxyl-chain-end –CH_2OH signal at δ=2.56 can be visible. Referring, in turn, to the ^{13}C NMR analysis (see Figure S2a among the signals characteristic for PVP, that is δ=179.5 ppm (C=O), δ=42.5-48.5 ppm (CH_2-CH-, the main chain), δ=33.9-37.7 ppm (-CH_2-CH-, the main chain), δ=31.8-32.9 ppm (O=C-CH_2-CH_2-CH_2-N-), δ=19.2 ppm (-CH_2-CH_2-CH_2-), a weak signal at 62.02 ppm assigned to hydroxyl-chain-end has appeared (CH_2OH). Moreover, it should be added that commercial PVPs can also be terminated with the hydroxyl group due to the presence of the water as a polymerizing medium and H_2O_2 initiator. This situation was observed, for example, in commercial PVP M_n=10.0 kg/mol [2]. Notably, this peak has not been visible in both the ^{1}H and ^{13}C NMR spectra of the commercial PVP K90, see Figure S1b and Figure S2b, respectively. We previously reported a similar result for the self-synthesized PVP in bulk [1].

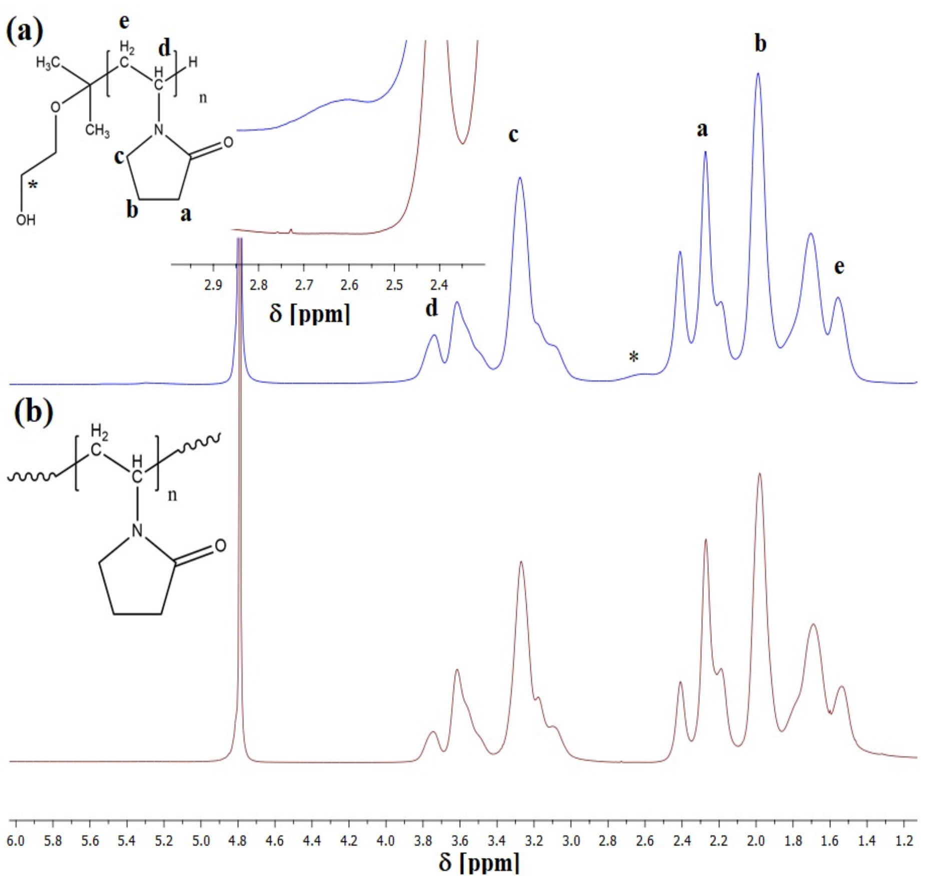

Figure S1. ^{1}H NMR spectra (D_2O) of (a) self-synthesized PVP-OH, and (b) commercial PVP K90.

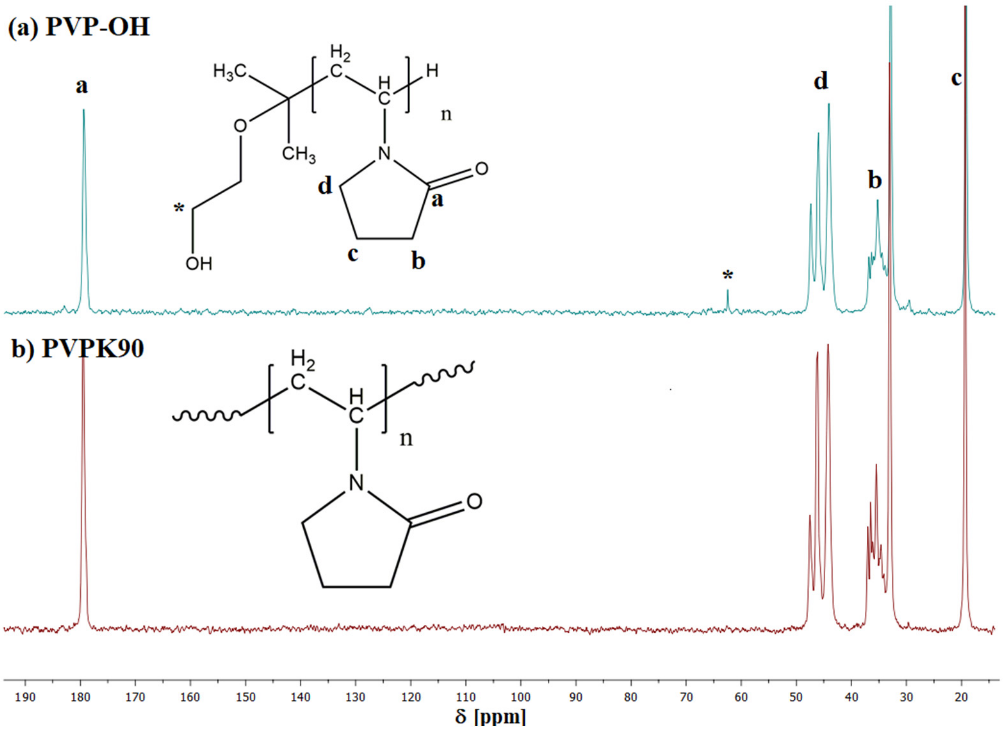

Figure S2. ^{13}C NMR spectra (D_2O) of (a) self-synthesized PVP-OH, and (b) commercial PVP K90.

^{13}C NMR assignments of triad and tetrad stereosequences

The PVP microstructure (tacticity) has been evaluated from the ^{13}C NMR analysis performed in D_2O. According to previous reports, the intensities of the different peaks were determined by peak deconvolution [3,4]. Note that D_2O solvent was applied due to more clearly separated signals than those visible in $CDCl_3$. The expanded carbonyl region allowed us to determine triad sequences (see Figure 2, main manuscript). In addition, based on the report [4], we thoroughly evaluated tetrad sequences from the β-methylene signals. As shown in Figure S3, the PVP K90 sample is dominated mainly by the r-centred tetrads (mrm, mrr), and such a phenomenon was previously reported for PVP prepared in aqueous solution [5]. In turn, PVP samples synthesized in bulk HP thermally-initiated methodology show similar tetrads sequences (irrespective of their M_n) on those reported for PVP K90, but with slightly higher content of isotactic fractions. This effect can directly result from the compression of the polymerizing mixture. More interesting, the PVP-OH sample, although prepared in a HP solvent-assistant FRP methodology, is mainly dominated by isotactic fractions with a relatively high content of syndiotactic ones, which, as we assume, can be a result of simultaneous action of both internal and external factors as a polar solvent and high-pressure, respectively.

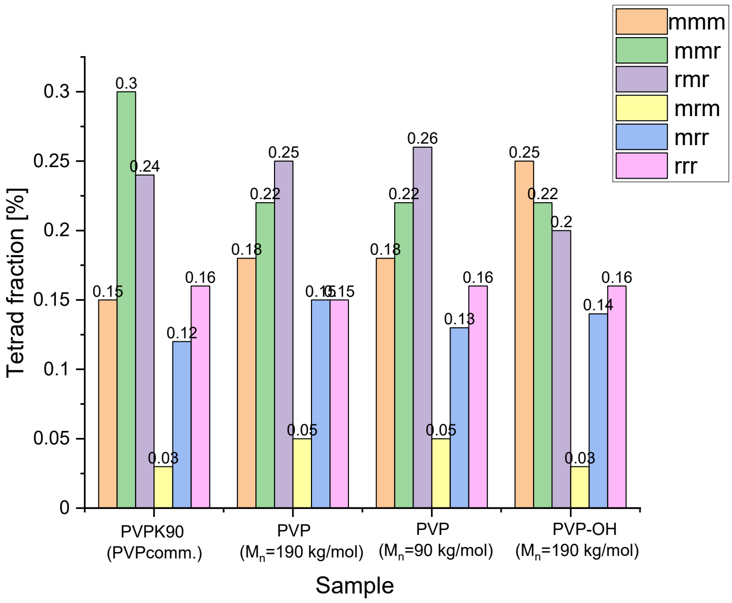

Figure S3. The content of tetrad fractions determined from β-methylene protons (^{13}C NMR, D_2O).

Size exclusion chromatography (SEC) analysis

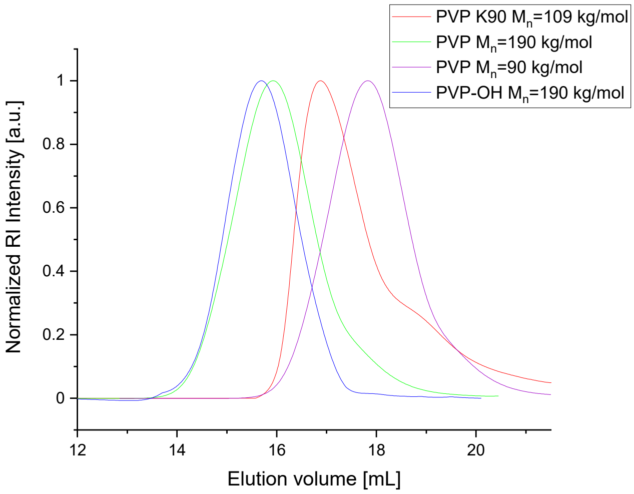

Figure S4. SEC traces of different PVPs used as matrices for FL (DMF+ 10mmol LiBr).

As shown in Figure S4, all self-synthesized PVPs obtained by the high-pressure methodology reveal symmetric and monomodal peaks shape demonstrate greater control over the polymerization process concerning the atmospheric pressure methodology. Moreover, in the case of the commercial PVP K90 sample, a high low-molecular-weight tail can be visible, proving the relatively high content of low M_n fraction in the synthesized sample. Dispersities of PVP K90 (M_n=109 kg/mol), PVP (M_n=190 kg/mol), PVP (M_n=90 kg/mol), and PVP-OH (M_n=190 kg/mol) took values Đ=1.78, Đ=1.86, Đ=1.47, Đ=1.63, respectively.

Non-isothermal calorimetric data

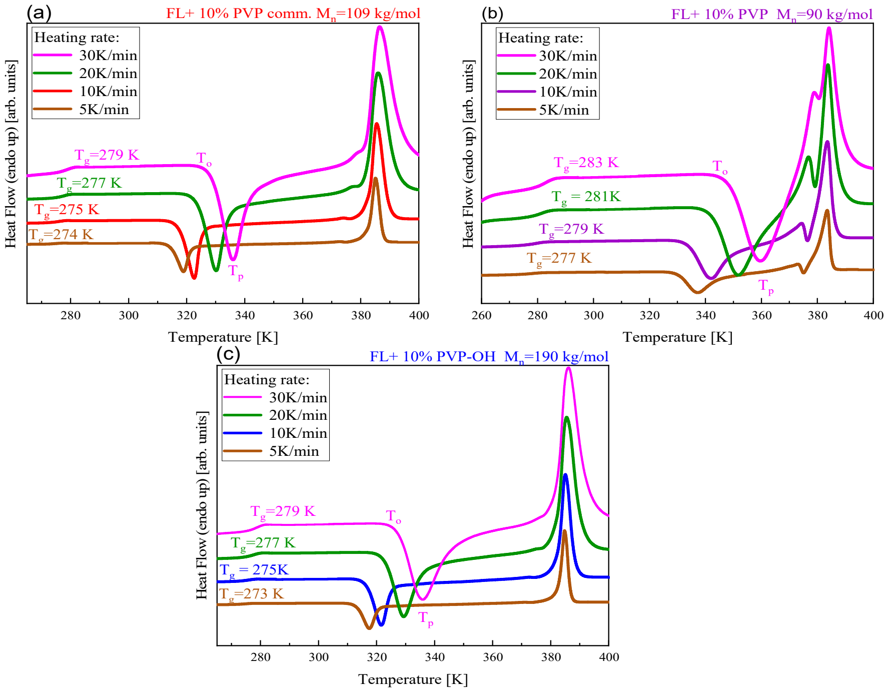

Figure S5. DSC curves obtained for binary mixtures of FL with PVPcomm. (a), PVP M_n=90 kg/mol (b) and PVP-OH M_n=190 kg/mol (c). Thermograms were measured with the indicated heating rates.

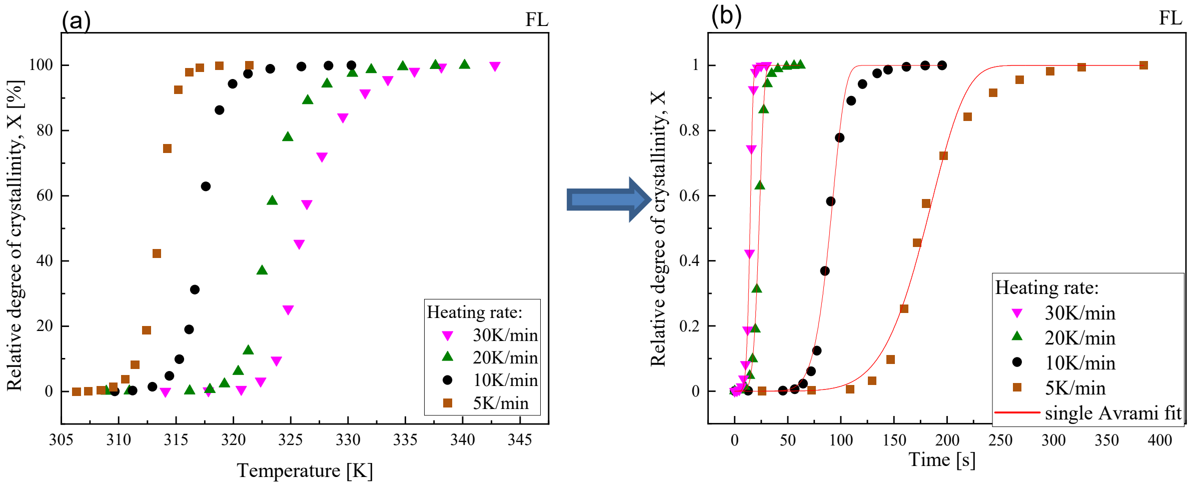

Figure S6. The relative degree of crystallinity obtained from Equation (4) at different ϕ, presented as a function of temperature (a). Time dependence of crystallization degree determined by transforming the temperature scale into the time scale using Equation (5) (b).

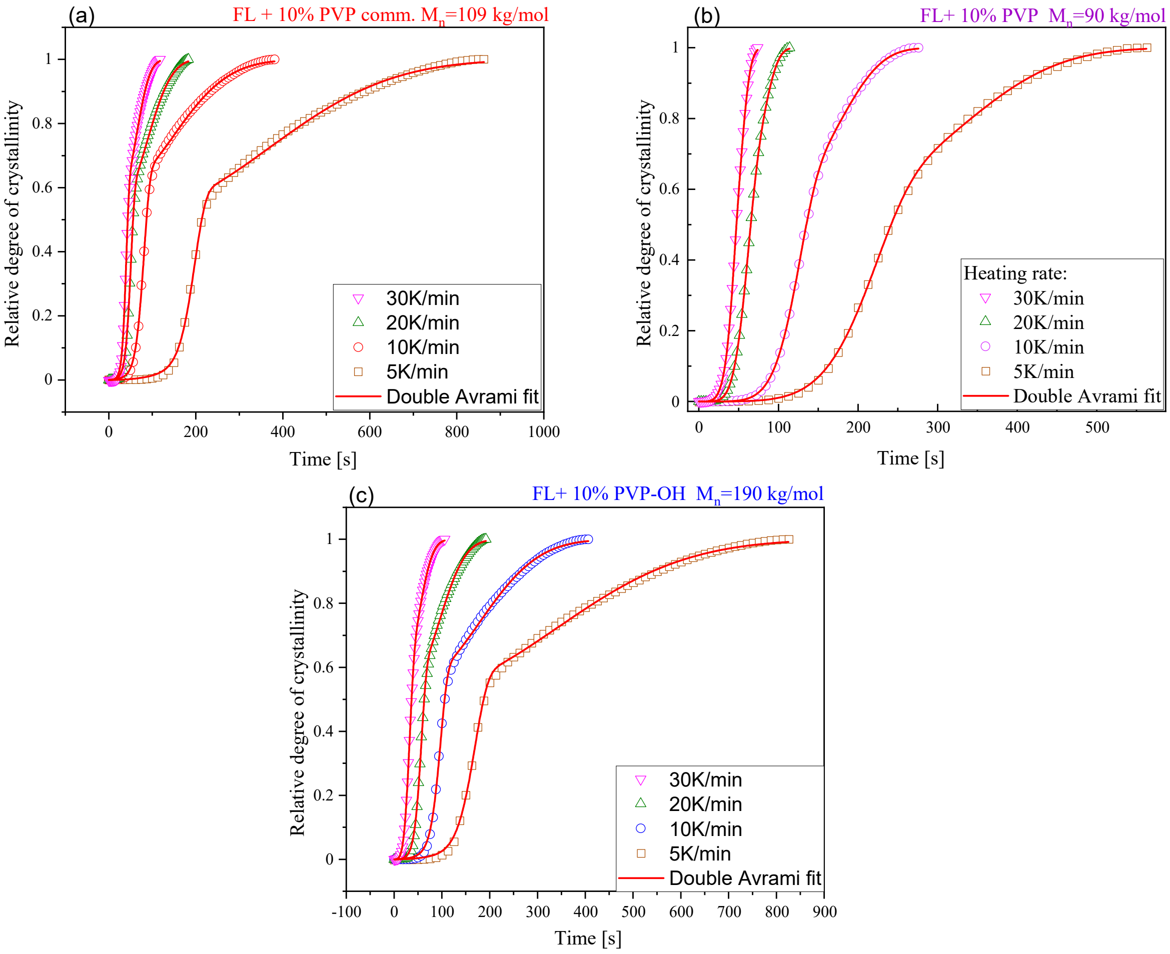

Figure S7. The relative degree of crystallinity versus time for binary mixtures of FL with 10 wt% of PVPcomm. (a), PVP M_n=90 kg/mol (b) and PVP-OH M_n=190 kg/mol (c). The solid lines represent double Avrami fits.

Infrared data

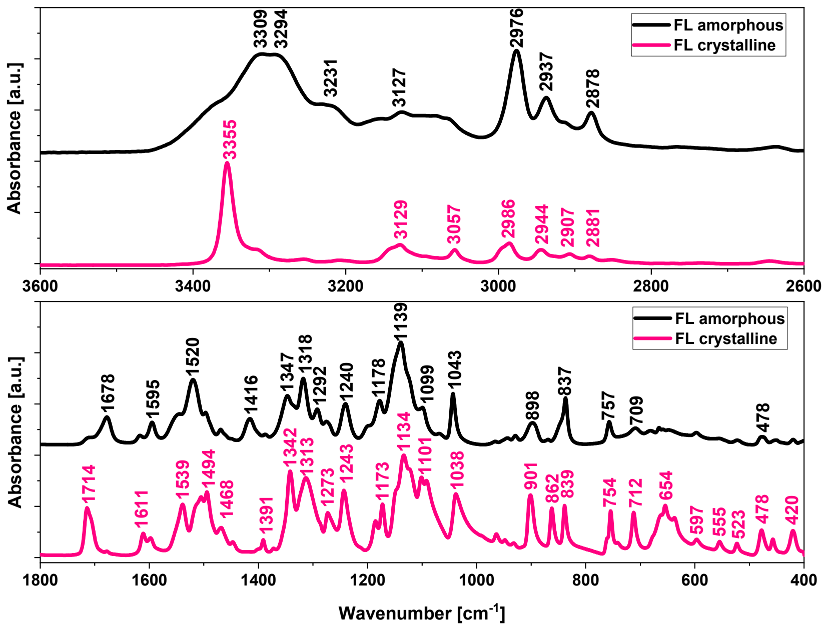

Figure S8. ATR-FTIR spectra of amorphous (red) and crystalline (black) FL samples presented in the high- and low-frequency ranges (3600-2600 cm^{-1} and 1800-400 cm^{-1}, respectively).

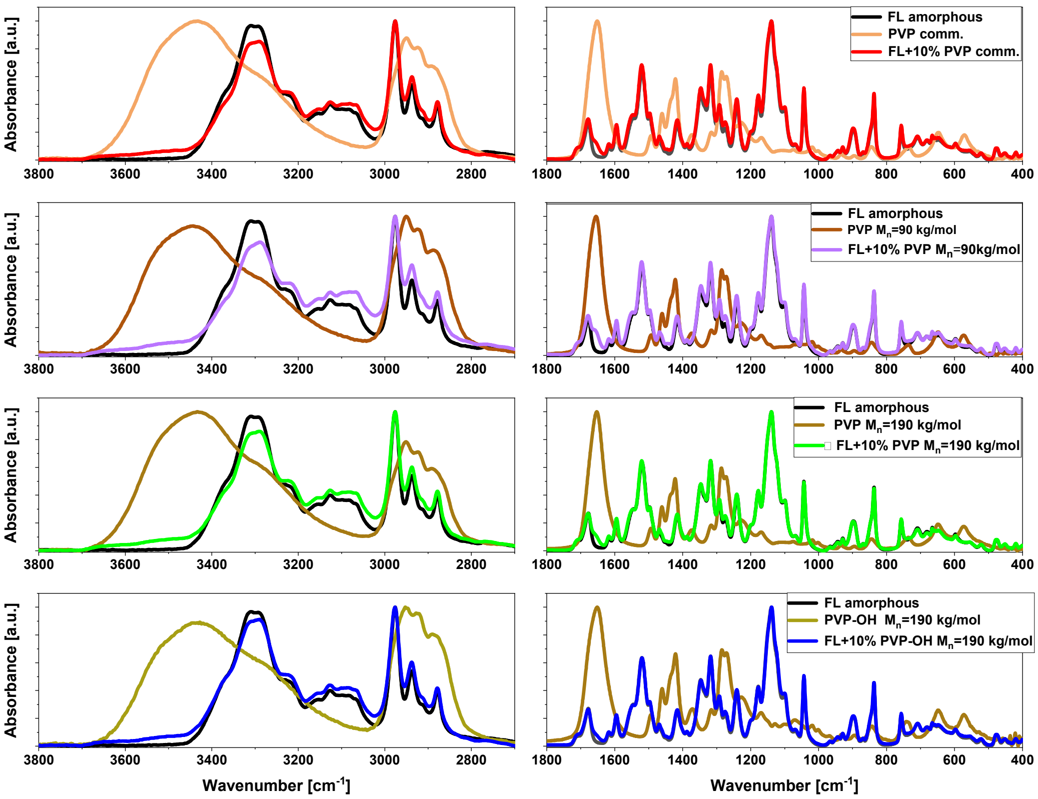

Figure S9. A comparison of the FTIR spectra of neat FL (black), PVP (red) as well as FL-polymer binary mixtures with 10% content of the excipient (blue) at 293K in the high- and low-frequency ranges (left and right, respectively).

References

1. Maksym, P.; Tarnacka, M.; Heczko, D.; Knapik-Kowalczuk, J.; Mielanczyk, A.; Bernat, R.; Garbacz, G.; Kaminski, K.; Paluch, M. Pressure-assisted solvent- and catalyst-free production of well-defined poly(1-vinyl-2-pyrrolidone) for biomedical applications. *RSC Adv.* **2020**, *10*, 21593–2160.

2. Washio, I.; Xiong, Y.; Yin, Y.; Xia, Y. Reduction by the end groups of poly(vinyl pyrrolidone): A new and versatile route to the kinetically controlled synthesis of Ag triangular nanoplates. *Adv. Mater.* **2006**, *18*, 1745–1749.

3. Hirano, T.; Miyamoto, Y.; Amano, S.; Tatsumi, K.; Anmoto, T.; Kimura, H.; Yoshida, K.; Oshimura, M.; Ute, K. Hydrogen-bond-assisted isotactic-specific radical polymerization of *N*-vinyl-2-pyrrolidone with tartrate additives in toluene at low temperatures: high-resolution 1H NMR analysis. *RSC Adv.* **2014**, *4*, 53079–53089.

4. Dutta, K.; Brar, A.S. Poly(vinylpyrrolidone): Configurational assignments by one- and two-dimensional NMR spectroscopy. *J. Polym. Sci. A Polym. Chem.* **1999**, *37*, 3922–3928.

5. Othmer, K. Encyclopedia of Chemical Technology, John Wiley & Sons, 2001, ISBN: 9780471484943, DOI: 10.1002/0471238961, R.B. Login, N-vinylamide polymers, p. 7.
